# Bee Stings

**Published:** 1871-01

**Authors:** 


Bee Stings.—A writer to the *Scientific American* says that "a good absorbent" will ease the pain of stings.

"The best absorbing substance that I have tried is lean fresh meat. This will relieve the pain of a wasp sting almost instantly, and has been recommended for the cure of rattlesnake bites. I have also used it with marked effect in erysipelas."

